# Low Concordance between Gene Expression Signatures in ER Positive HER2 Negative Breast Carcinoma Could Impair Their Clinical Application

**DOI:** 10.1371/journal.pone.0148957

**Published:** 2016-02-19

**Authors:** Enora Laas, Peter Mallon, Francois P. Duhoux, Amina Hamidouche, Roman Rouzier, Fabien Reyal

**Affiliations:** 1 Institut Curie, Department of Surgery, Paris, France; 2 Hopital Tenon, Department of Gynaecologic Surgery, Paris, France; 3 Institut Curie, Department of Medical Oncology, Paris, France; 4 Centre du Cancer, Cliniques universitaires Saint-Luc, Université catholique de Louvain, B-1200 Brussels, Belgium; 5 Institut Curie, Translational Research Department, Residual Tumor and Response to Treatment, RT2Lab, Paris, France; 6 Institut Curie, UMR932, Immunity and Cancer, Paris, France; 7 Craigavon Area Hospital Breast Unit, Portadown Northern Ireland, BT63 5QQ; University of North Carolina School of Medicine, UNITED STATES

## Abstract

**Background:**

Numerous prognostic gene expression signatures have been recently described. Among the signatures there is variation in the constituent genes that are utilized. We aim to evaluate prognostic concordance among eight gene expression signatures, on a large dataset of ER positive *HER2* negative breast cancers.

**Methods:**

We analysed the performance of eight gene expression signatures on six different datasets of ER+ HER2- breast cancers. Survival analyses were performed using the Kaplan–Meier estimate of survival function. We assessed discrimination and concordance between the 8 signatures on survival and recurrence rates The Nottingham Prognostic Index (NPI) was used to to stratify the risk of recurrence/death.

**Results:**

The discrimination ability of the whole signatures, showed fair discrimination performances, with AUC ranging from 0.64 (95%CI 0.55–0.73 for the 76-genes signatures, to 0.72 (95%CI 0.64–0.8) for the Molecular Prognosis Index T17. Low concordance was found in predicting events in the intermediate and high-risk group, as defined by the NPI. Low risk group was the only subgroup with a good signatures concordance.

**Conclusion:**

Genomic signatures may be a good option to predict prognosis as most of them perform well at the population level. They exhibit, however, a high degree of discordance in the intermediate and high-risk groups. The major benefit that we could expect from gene expression signatures is the standardization of proliferation assessment.

## Introduction

Multi-gene expression assays as tools for clinical decision-making are increasingly being used in clinical practice. The 2011 St. Gallen International Expert Consensus [1] recognized the 21-gene signature (Oncotype DX, GenomicHealth) as a tool that "may be used" to predict chemotherapy responsiveness in patients with ER positive node negative breast tumors. The latest NCCN guidelines [2] include "multi-gene testing" in the list of factors used to select local and systemic therapies. They stated that the 21-gene signature may assist in evaluating patients for chemotherapy whose primary invasive breast tumors are 0.6–1.0 cm with unfavourable features or >1 cm and node-negative, ER/PR positive and HER2-negative.

The 21-gene assay (Oncotype DX), uses reverse transcription polymerase chain reaction (RT-PCR) on RNA isolated from paraffin-embedded breast cancer tissue. Two retrospective trials on Oncotype DX have quantified the risk of recurrence as a continuous variable and can predict response to tamoxifen [3] and chemotherapy [4] in patients with ER positive node negative disease. The Oncotype DX recurrence score assay also provides predictive information for chemotherapy benefit in patients with axillary lymph node-positive ER-positive breast cancer. The data in node-positive patients, however, was less robust as it relied on retrospective subset analysis of a single randomized trial [5].

The MammaPrint assay (70-gene expression profile) is approved by the FDA to assist in categorizing women younger than 61-years into a high vs. low risk of recurrence [6,7]. The Mindact trial^8^ will help conclude if Mammaprint can assist in selection of adjuvant chemotherapy for breast cancer patients with 0–3 nodes

Other multi-gene expression assay systems are currently not recommended for use in clinical practice but there is an increasing amount of literature reporting on their clinical relevance [8–14].

Previous studies have however raised concerns about concordance and stability of gene signatures for ER/PR/HER2 negative[15] and ER positive/HER2 negative cancers[16,17].Another concern is that expression levels of single and multi-gene signatures are unstable[18–20] In a previous study, we found that the concordance of outcome assignment between eight gene expression signatures was low [20]. Furthermore, a recent review by Engelhardt et al showed that the accuracy of genes signature prognostic estimates was suboptimal in some patient subgroups [21]. These signatures were designed to help oncologists make clinical decisions on the necessity of adjuvant therapy. It is important, therefore, to determine if current signatures are standardized and reproducible in different patient sub-groups.

In this study, we aim to evaluate the concordance in survival prediction of 8 gene-expression signatures on a large dataset of ER positive *HER2* negative breast cancers.

## Materials and Methods

### Data pre-processing

As previously described, six breast cancer datasets [9,22–26] (all arrayed on the same platform [HGU-133A Affymetrix Santa Clara, California, USA] to avoid cross-platform discrepancies) for which the raw data (.CEL files) were publicly available were downloaded from the Gene Expression Omnibus and ArrayExpress repository websites [27,28]. This resulted in a total of 1,143 microarrays. Of these, 1,127 were deemed to be of sufficient quality and were kept in the current study. Sixteen micro-arrays were rejected due to major artifacts in the original.CEL files. To ensure comparability between the different datasets, they were all subjected to the same pre-processing procedure. Microarray quality-control assessment was carried out using the AffyPLM R-package [29]. Selected arrays were normalized using the RMA expression measure algorithm [30].

### Determination of ER/Her2 status

The oestrogen receptor (ER) gene expression status was determined using the 205225_at probe set [31]. A gene-expression cut-off of 1.834 resulted in a 90% sensitivity in determining the ER status (IHC). All samples with a gene expression value higher than 1.834 were classified as ER positive. Similarly, the Her2 gene expression status was determined using the 216836_s_at probe set. A density plot of the 1,127 gene expression values showed a bimodal distribution. The lowest value of the density plot between the two modes determined the cut-off between the Her2 positive and Her2 negative status. All samples with a gene expression value higher than 1.62 were classified as Her2 positive.

### Signature validation

On the complete dataset, we applied the 76-gene signature [8]; Chromosomal Instability Signatures [CIN70, CIN25] [11]; Core Serum Response signature [CSR] [12]; Invasiveness Gene Signature [IGS][13]; Molecular Prognosis Index signature [T52, T17] [10] and Gene expression Grade Index [GGI] [14]. These classifiers were defined as previously described[20].

### Statistical analysis of the concordance of the 8 gene signatures

To identify robust subgroups of patients with different outcome we used the Nottingham Prognostic Index (NPI) score. It is based on a combination of three pathological criteria (tumour size, lymph-node involvement stage and tumour grading) assembled in a prognostic index formula [32]. It can be used as a continuous variable for risk stratification in unselected cohorts of operable early-stage primary breast cancer patients. Prognosis worsens as the NPI numerical value increases. It can also stratify patients into good, moderate and poor prognostic groups [33]. The NPI has been validated independently in large multicentre studies [34,35]. Because the precise number of positive axillary nodes was not known, we used a slightly different definition for the NPI, as described by Teschendorff et al [36]: NPI = 0.2 × (Tumor_Size [cm]) + Grade + 1.5 × (Node_Status) + 1 where Node Status can be positive (1) or negative (0) and Grade can be 1, 2 or 3. Scores lower than 3.4 defined the low risk group. A score between 3.4 and 5.4 defined intermediate risk group, whereas the high-risk group had a score higher than 5.4.To evaluate signatures performance, we performed survival analyses using the Kaplan–Meier estimate of the survival function. Comparison between survival curves was performed using the log-rank test. P values were considered significant when <0.05. Discrimination (i.e., whether the relative ranking of individual predictions was in the correct order) was quantified in both populations with the measure of the area under curve (AUC) ROC. For this analyze, the outcome was dichotomized into a poor outcome group (samples with an event within 5 years of follow-up) and a good outcome group (samples with no event and a follow-up of at least 5 years. Events were defined as distant metastases occurrence or breast cancer specific death. Concordance was first strictly defined as 0 or 8 poor prognosis signatures. Then, we observed concordance with a broader definition: 0 or 1 and 7 or 8 poor signatures.

We observed the distribution of discordant signatures predictions, according to the NPI stratification (low risk, intermediate risk, high risk).

The analysis was performed using R software using the *survival*, *rms and pROC* package (http://cran.r-project.org/).

## Results

### Population

From our previous study, we identified 769 patients out of the 1,127 presenting an ER positive HER2 negative breast carcinoma. For 454 samples, distant metastasis-free survival (DMFS) data were available; while for 349 samples, breast cancer-specific survival (BCSS) data were available. The clinical and pathological features are described in Table 1.

**Table 1:** Clinical, histological and molecular signatures of 769 ER-positive HER2-negative patients presenting breast carcinoma^*^.

	Distant-Metastasis Free Survival(n = 454)	Breast Cancer Specific Survival(n = 349)
**Tumour Size**		
pT1 (%) (≤20mm)	213 (47)	234 (67)
Median (min-max)	22 (3–100)	20 (2–70)
**Histological Grading. Elston Ellis**		
Grade I (%)	97 (21)	92 (26)
Grade II (%)	203 (45)	173 (49)
Grade III (%)	76 (17)	71 (20)
Not available (%)	78 (17)	13 (3.7)
**Lymph Node Metastasis**		
Positive (%)	138 (30)	91 (26)
Not Available (%)	7 (1.5)	116 (33)
**Distant Metastasis or Death from Breast Cancer**		
Positive (%)	109 (24)	61 (17)
Within 5 years	70 (15.4)	11 (3.1)
**Follow-Up**		
Median (min-Max). Months	85 (0–293)	80 (0–154)

*Data on DMFS and BCSS are not available for all patients

### NPI Prognostic performance

**Fig 1:**
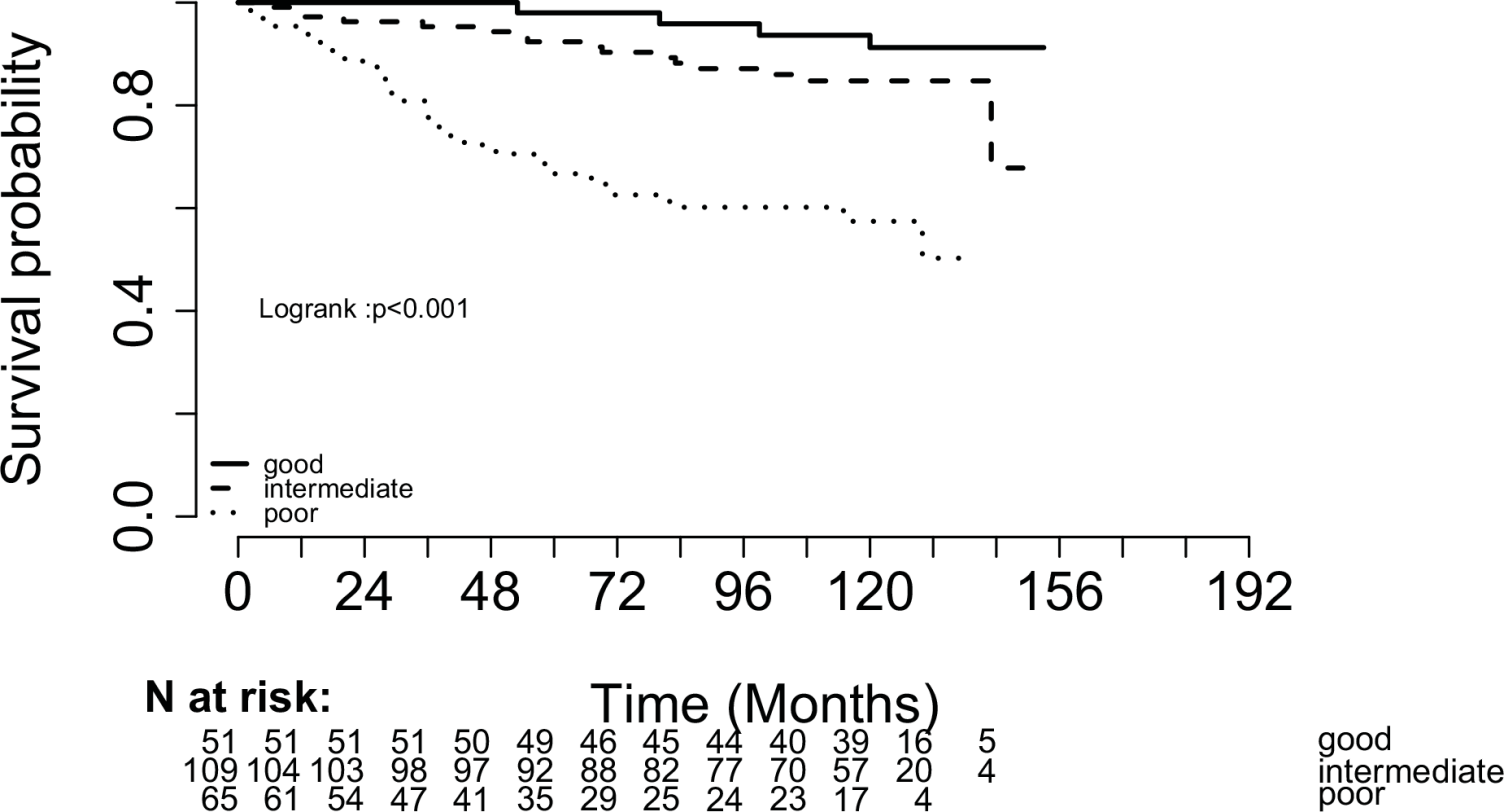
Breast Cancer Specific Survival according to the Nottingham Prognosis Index.

The NPI score was able to identify in our population three patients subgroups with significantly different outcome. 22.5% were classified as low risk (13.2% of events), 57.3% were classified as intermediate risk (21.7% of events) and 20.2% were classified as high risk (36.7% of events). The 5 years survival rate (BCSS and DMFS) was significantly different between groups (Log rank pvalue <0.001): 98% in the low-risk group, 92% in the intermediate-risk group (HR 4.67) and 66.7% in the high-risk group (HR 20.03) (Fig 1).

### Gene expression Signatures performance

The percentage of patients with a “poor prognosis” call for each signature varies between 30 and 68%.

**Fig 2:**
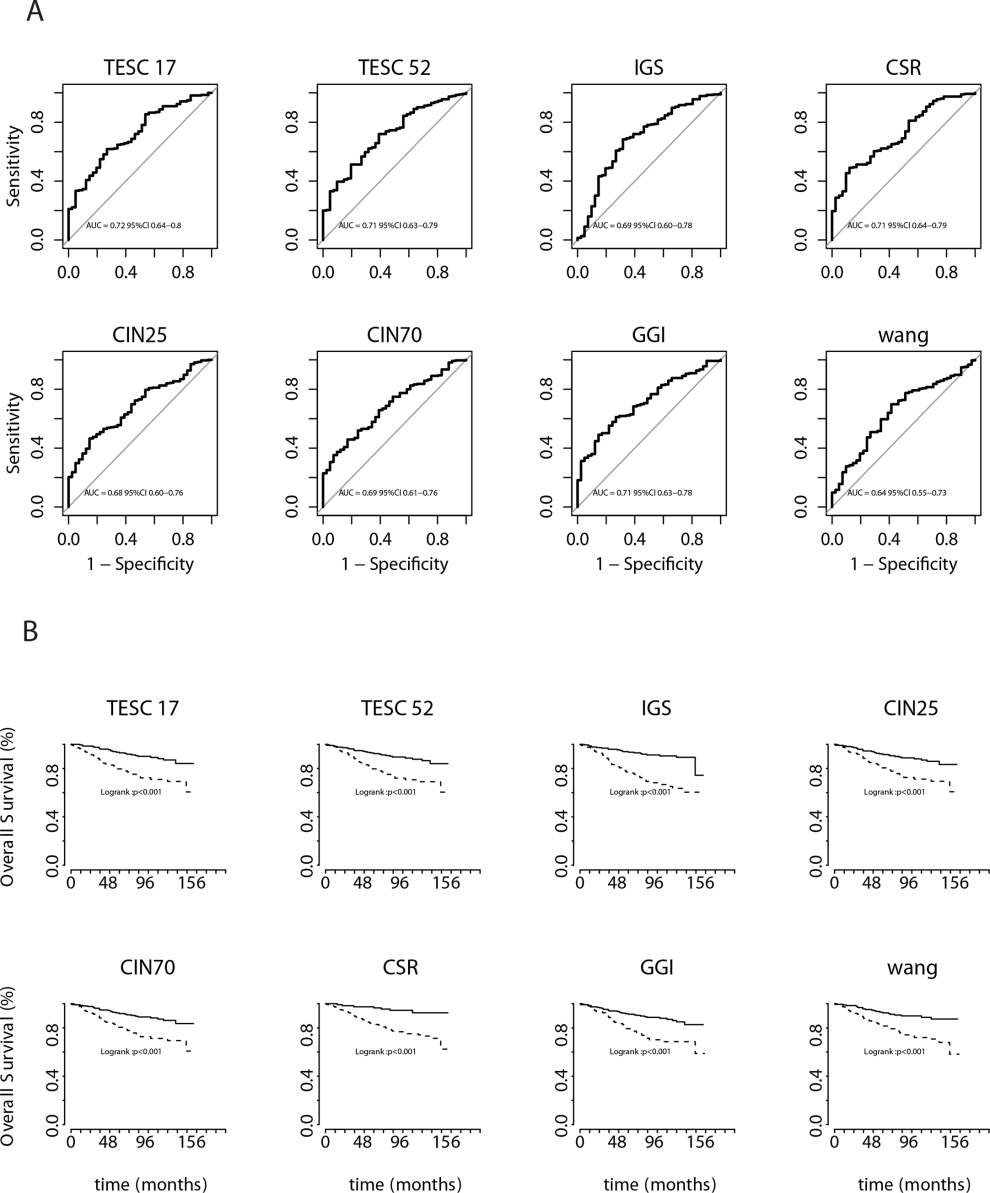
Performance of the eight molecular signatures (A)Breast Cancer Specific Survival (Kaplan Meier curves) (B) Discrimination performances (ROC curves)

For the BCSS, the performance in terms of log-rank test p-value was similar between the different molecular signatures (Fig 2A), with hazard ratios ranging from 2.4 for the CIN25, CIN70 and GGI signatures to 4.5 for the CSR signature (Table 2).

**Table 2:** Gene expression signatures performance in the prediction of Distant-Metastasis Free Survival and Breast Cancer Specific Survival^*^.

	Distant-Metastasis Free Survival(n = 454)	Breast Cancer Specific Survival(n = 349)
**T52 signature**		
Poor prognosis (%)	175 (38)	131 (37)
Cox model. Hazard Ratio (95% CI). p.value	1.6 (1.2–2.3) p = 0.004	2.6 (1.6–4.3) p = 0.0001
**T17 signature**		
Poor prognosis (%)	197 (43)	143 (41)
Cox model. Hazard Ratio (95% CI). p.value	1.7 (1.2–2.4) p = 0.001	2.7 (1.6–4.5) p<0.001
**IGS signature**		
Poor prognosis (%)	182 (40)	125 (36)
Cox model. Hazard Ratio (95% CI). p.value	2.2 (1.6–3) p<0.001	3.8 (2.2–6) p<0.001
**CSR signature**		
Poor prognosis (%)	312 (68)	229 (65)
Cox model. Hazard Ratio (95% CI). p.value	1.6 (1.1–2.4) p = 0.01	4.5 (2–9) p<0.001
**CIN70 signature**		
Poor prognosis (%)	178 (39)	131 (37)
Cox model. Hazard Ratio (95% CI). p.value	1.5 (1.1–2.1) p = 0.01	2.4 (1.4–4) p<0.001
**CIN25 signature**		
Poor prognosis (%)	177 (39)	125 (36)
Cox model. Hazard Ratio (95% CI). p.value	1.6 (1.1–2.1) p = 0.009	2.4 (1.4–4) p<0.001
**GGI signature**		
Poor prognosis (%)	157 (35)	110 (32)
Cox model. Hazard Ratio (95% CI). p.value	1.8 (1.3–2.5) p<0.001	2.4 (1.4–4) p<0.001
**Wang signature**		
Poor prognosis (%)	235 (52)	154 (44)
Cox model. Hazard Ratio (95% CI). p.value	1.4 (1–2) p = 0.03	2.7 (1.6–4.7) p<0.001

*Data of DMFS and BCSS are not available for all patients

For the DMFS, the performance was also similar between the different molecular signatures with hazard ratios ranging from 1.4 for the Wang signature to 2.2 for the IGS signature (Table 2).

Similarly, the discrimination ability of the whole signatures for both DMFS and BCSS, measured using the AUC, showed fair discrimination performances. The AUC ranged from 0.64 for the Wang signature to 0.72 for the TESC17 signature, with overlapped confidence interval (Fig 2B).

However, from an individual viewpoint, 39.9% of the events failed to be predicted by none or less than 5 signatures (Table 3).

**Table 3:** Repartition of patients according to number of poor signature.

Number of poor signature	Patients		Events*	
	n	%	n	%
0	198	25.7	13	8.1
1	138	17.9	22	13.7
2	76	9.9	16	9.9
3	40	5.2	12	7.5
4	23	3.0	4	2.5
5	32	4.2	9	5.6
6	27	3.5	3	1.9
7	60	7.8	20	12.4
8	175	22.8	62	38.5

*Distant metastasis or death from breast cancer

### Signature concordance and accuracy

When concordance was defined as none (0) or 8 signatures with similar outcome assignments, more than 50% of the population (51.5%) had a discordant prediction. 53% of all events were identified in this subgroup (Table 3). The discordance rates were similar between the three risk subgroups as defined by the NPI score: low-risk group 52.1%, intermediate-risk group 53.1% and high-risk group 51.4% (p = 0.9).

**Fig 3:**
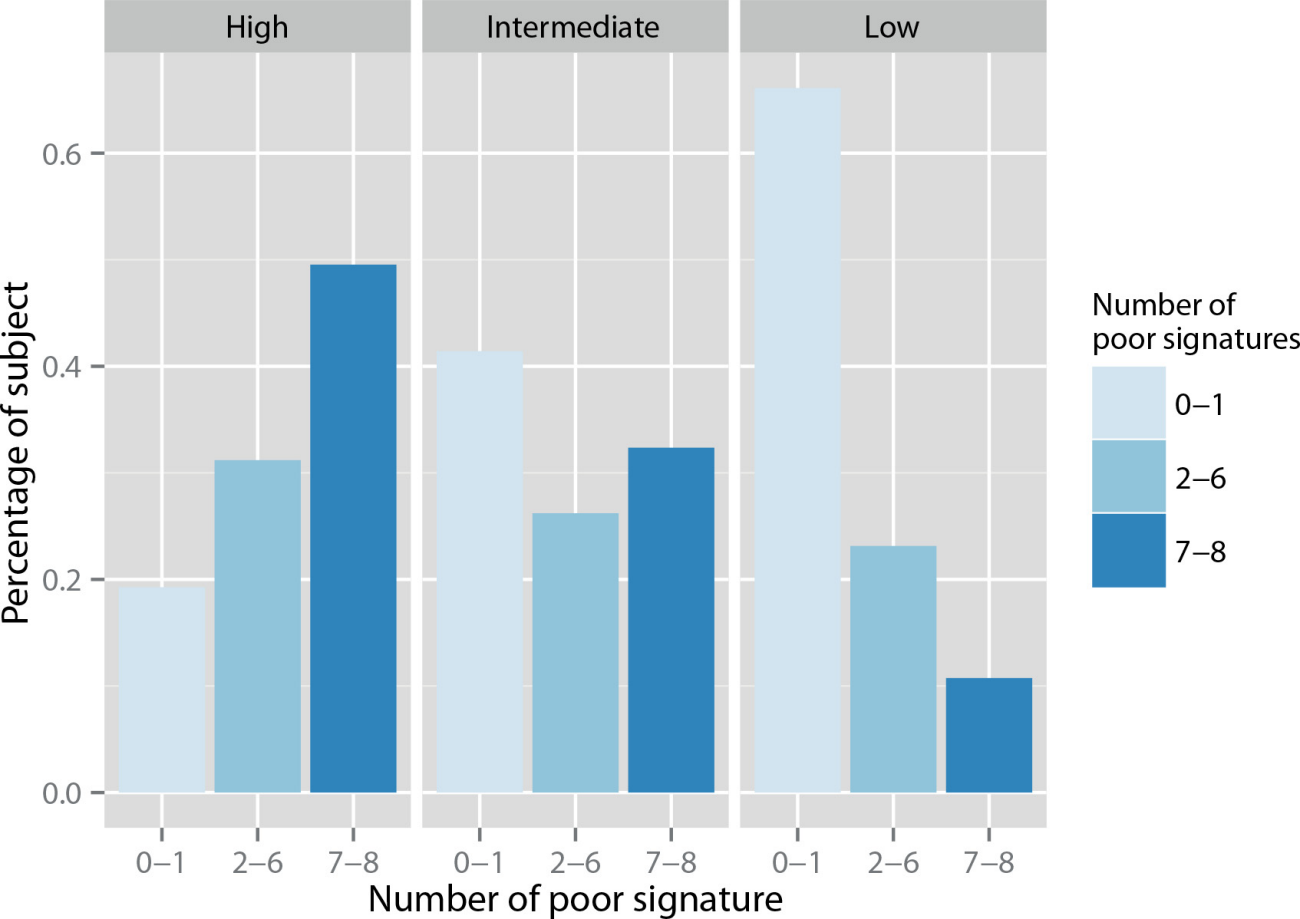
Concordance of the 8 molecular signature, according to the modified-NPI risk groups*. *We considered concordance for 0 or 1 signature and 7 or 8 signatures.

With a broader concordance definition (0–1, 7–8 poor outcome signatures), 25.8% of the population had a discordant prediction, with only 2 to 6 signatures with similar outcome assignments. 27.4% of all events were identified in this subgroup (Table 3). The low-risk group, as defined by a NPI score below 3.4, had the highest concordance between signatures, with 66.1% of concordant good prognosis prediction (0 or 1 poor signature), and 23.1% of discordant signatures (2 to 6). The discrepancy rate was higher in the intermediate and high-risk subgroup, with 26.2% and 31.2% of signatures discordances respectively (p = 0.4) (Fig 3).

### Impact of gene-signature number on concordance

**Fig 4:**
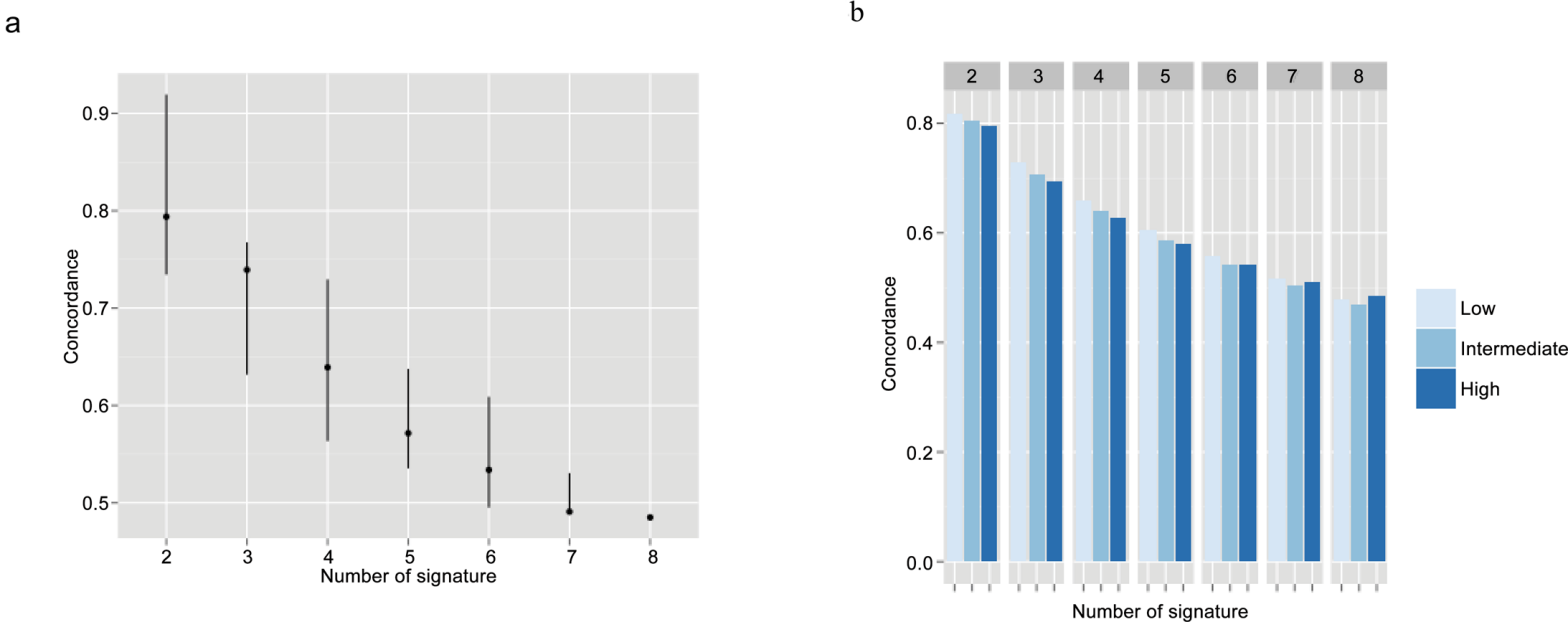
Concordance of the prediction with molecular signatures combination. **Combination of 2 to 7 molecular signatures.** (A)Overall population (B) NPI risk groups.

We calculated the concordance for every combination of 2 (28 combinations), 3 (56 combinations), 4(70 combinations), 5 (56 combinations), 6 (28 combinations), and 7 (8 combinations) gene-expression signatures out of the 8 available in this study. The median concordance rate decreased significantly with the number of signature available: from 79.4% for 2 signatures to 48.5% if we used 8 signatures (Fig 4A). Regardless of the number of signatures available (2 to 8), the concordance was consistently better in the low-risk group than in the intermediate-risk group and high-risk group (p value = 1) (Fig 4B).

## Discussion

We observed that 8 gene signatures consistently identified a small subgroup of ER-positive HER2-negative breast tumors patients with an excellent prognosis. In this subgroup, the risk of distant events remains low (4–6%) and would eventually justify a systemic treatment with tamoxifen or aromatase inhibitors alone. In addition, the 8 gene signatures consistently identified a small subgroup of patients with the worst prognosis. This however did not translate to clinical outcome, as 65% of this patient group did not develop distant metastases, or disease specific death. Between these two poles of high concordance we identified a large “grey zone” were gene-expression signatures did not reach a consensus in the prediction of events. The discordance increased with the number of signature used. This heterogeneity may be attributed to the different methodologies that were followed to build the classifiers, the heterogeneity in the sample populations used to build the classifiers, and the variation in sample size s[37]. Many genes signatures exist and many more will be available over the years with little agreement in the constituent genes. Wirapati et al showed that the prognostic abilities of nine gene expression signatures would be due mostly to the detection of proliferation activity [15]. However, few studies have investigated the concordance between existent predictors. Fan et al have shown significant agreement (81%) in the outcome predictions of five genomic signatures (including Mammaprint and OncotypeDX), pairwise compared [38]. In another study from Haibe-Kains et al, similar prognostic performances were found between the 70-gene, the 76-gene and the Gene expression Grade Index signatures (68% when considering the three signatures, 71% for the 70- and 76-gene signatures, 76% for the 76-gene signature and the GGI, and 88% for the 70-gene signature and the GGI). [39].

Several studies have described methods to improve the stability of gene signatures. One way is to increase the sample size. It generates a gene signature with better concordance in outcome prediction and better prediction accuracy [40]. Other statistical methods (resampling, bootstrap) can improve stability and performance in some cases. Another way of improving the stability of a gene signature suggested by Zhao et al was to combine the information obtained from published gene-sets prognosis signatures to build a higher prognostic performance signature using an independent gene expression dataset [41]. In a previous study, Reyal et al showed that the combination of the signatures could define an efficient classifier for breast cancer, which will most probably be more stable than the classifiers from which it originates [20].

It is therefore not surprising that the risks predicted by these models are in the end little used in clinical setting for adjuvant therapies prescription. In the review from Hornberger et al, use of risk prediction model (comprising Oncotype DX, Mammaprint or Adjuvant Online) led to a change in treatment recommendation ranging from 1% to 74% [42], but with considerable heterogeneity between studies.

It is thus debatable whether these expensive tools add significant information to the clinical and biological data already available,. The use of Ki67 may increase in popularity as one of its major drawbacks, the lack of standardization, has been addressed by recent international recommendations [43]. Refining outcome prediction by adding other immunohistochemical data like TP53 expression is another route that should be explored and that might prove more cost-effective than the use of gene expression signatures [44].

New models based on genetic profiles combined with clinical factors, may further improve prediction. Tang et al have associated the OncotypeDx recurrence score to clinicopathologic factors in the Recurrence Score Pathology-Clinical (RSPC) score. This model substantially reduces the number of patients classified in the intermediate risk category in the assessment of distant recurrence risk [45]. Similarly, the immunohistochemical (IHC) 4+C score is a cost-effective prognostic tool that uses clinicopathologic factors and four standard IHC assays: oestrogen receptor, progesterone receptor, HER2 and Ki67 [46]. It has been shown to substantially improve decision-making on adjuvant chemotherapy [47]. Obviously, concordance of these predictions should be evaluated too, since improvement of prediction does not mean improvement in concordance of prediction.

One of the major limitations of our study is to not integrate the two well-known signatures that are currently the most widely used and validated: the 21-gene Genomic Health signature OncotypeDX [3] and the 70-gene signature from the Netherlands Cancer Institute (Mammaprint) [7]. The Genomic Health signature was not included since it is not a micro-array-based gene expression signature but is designed for RT-PCR assays. The 70-gene signature was not included since the 295 samples dataset on which this signature was developed was employed as a completely independent validation set.

In conclusion, regarding the adverse events and costs of adjuvant therapies, reliable decision assisting tools must be developed. Genomic signatures could be a good option, but this study revealed that if most genomic signatures perform well at the population level, they exhibit a high degree of discordance in the intermediate and high-risk groups. Moreover, in the high-risk group, there were only 38.5% of events when the 8 signatures were concordant for a poor prognosis, adding to the measurement error. Clinical as well as genomic prediction models in ER positive *HER2* negative breast cancer subgroups are only able to reliably identify good prognosis patients. The main argument to support the use of genomic predictors would be a single standardized gene signature tool. Unfortunately, the current high level of discordance between gene signatures complicates their use for routine clinical practice. We have demonstrated in this study that the use of multiple genomic signatures in ER-positive HER2-negative breast tumours affects the accuracy of prognosis prediction.
